# Development and internal validation of a machine learning prediction model for low back pain non-recovery in patients with an acute episode consulting a physiotherapist in primary care

**DOI:** 10.1186/s12891-022-05718-7

**Published:** 2022-09-03

**Authors:** J. Knoop, W. van Lankveld, L. Beijer, F. J. B. Geerdink, M. W. Heymans, T. J. Hoogeboom, S. Hoppenbrouwers, E. van Overmeeren, R. Soer, C. Veenhof, K. C. P. Vissers, P. J. van der Wees, M. Sappelli, J. B. Staal

**Affiliations:** 1 Musculoskeletal Rehabilitation Research Group, HAN University of Applied Sciences, PO Box 6960, 6503 GL Nijmegen, Netherlands; 2 Research and Innovation Department, Sint Maartenskliniek, Nijmegen, Netherlands; 3 Research Group Smart Health, Saxion University of Applied Sciences, Enschede, Netherlands; 4 Department of Epidemiology and Data Science, Amsterdam UMC, Amsterdam, Netherlands; 5 Radboud Institute for Health Sciences, Radboud University Medical Centre, IQ Healthcare, Nijmegen, Netherlands; 6 Academy of IT and Mediadesign, Data and Knowledge Engineering Research Group, HAN University of Applied Sciences, Nijmegen, Netherlands; 7 Institute for Computing and Information Sciences, Radboud University, Nijmegen, Netherlands; 8 Royal Dutch Society for Physical Therapy, Amersfoort, Netherlands; 9 University of Groningen, University Medical Center Groningen, Groningen Pain Center, Groningen, Netherlands; 10 Department of Rehabilitation, Physiotherapy Science and Sport, University Medical Center Utrecht, Utrecht, Netherlands; 11 Department of Anesthesiology, Pain and Palliative Medicine, Radboud University Medical Center, Nijmegen, Netherlands

**Keywords:** Low back pain, Acute, Recovery, Prognostic, Machine learning

## Abstract

**Background:**

While low back pain occurs in nearly everybody and is the leading cause of disability worldwide, we lack instruments to accurately predict persistence of acute low back pain. We aimed to develop and internally validate a machine learning model predicting non-recovery in acute low back pain and to compare this with current practice and ‘traditional’ prediction modeling.

**Methods:**

Prognostic cohort-study in primary care physiotherapy. Patients (*n* = 247) with acute low back pain (≤ one month) consulting physiotherapists were included. Candidate predictors were assessed by questionnaire at baseline and (to capture early recovery) after one and two weeks. Primary outcome was non-recovery after three months, defined as at least mild pain (Numeric Rating Scale > 2/10). Machine learning models to predict non-recovery were developed and internally validated, and compared with two current practices in physiotherapy (STarT Back tool and physiotherapists’ expectation) and ‘traditional’ logistic regression analysis.

**Results:**

Forty-seven percent of the participants did not recover at three months. The best performing machine learning model showed acceptable predictive performance (area under the curve: 0.66). Although this was no better than a’traditional’ logistic regression model, it outperformed current practice.

**Conclusions:**

We developed two prognostic models containing partially different predictors, with acceptable performance for predicting (non-)recovery in patients with acute LBP, which was better than current practice. Our prognostic models have the potential of integration in a clinical decision support system to facilitate data-driven, personalized treatment of acute low back pain, but needs external validation first.

**Supplementary Information:**

The online version contains supplementary material available at 10.1186/s12891-022-05718-7.

## Introduction

Most people experience an episode of acute low back pain (LBP) at some point in their life [1], and in at least 85% of the cases this pain is labelled as ‘non-specific LBP’ (i.e., no patho-anatomical cause of the symptoms identified) [2]. LBP is the leading cause of disability worldwide and accompanied by high health care utilization and societal costs [1], of which the majority can be attributed to those patients in the chronic phase of LBP (i.e., symptoms > three months) [2]. It is therefore very important to identify those patients with acute LBP who are at risk for chronic LBP, in order to potentially prevent the transition from acute to chronic LBP and the associated costs.

The course of LBP over time is considered highly heterogeneous and the underlying mechanisms are not yet fully understood [1]. Current beliefs hold that the majority of patients with acute LBP recover within 3 months, while those who do not are very likely to suffer from chronic LBP for many years [3]. However, the course of acute LBP symptoms appears to be far more complex: on the one hand, in many people with acute LBP, there symptoms reduce substantially within the first month [4], while on the other hand, a large majority of them will be faced with a LBP recurrence within twelve months [5].

In the past decades, a plethora of studies have been conducted to link potential predictors such as biological, psychological and social/occupational factors to LBP (non-)recovery. Based on a number of systematic reviews [2, 6–9], only a limited number of factors could be consistently identified as predictors, while conflicting evidence was found for the majority of these predictors. Moreover, individual factors, even if they have consistently been found to be a predictor, will have only little prognostic value on their own, and should be combined with other predictors for an accurate prediction [3].

Health care providers are generally unable to adequately predict the course of acute LBP based on their clinical expertise [10]. Therefore, in the last decade, a number of prognostic tools (e.g. [11–14],) have been developed to guide health care providers in their clinical decision making process. This may improve clinical outcomes while also preventing unnecessary care in acute LBP [15]. The current most frequently used tool among physiotherapists in LBP is the STarT Back screening Tool (SBT) [11]. Although the SBT has been found to be valid and reliable for distinguishing low, medium and high risk profiles, this was predominantly tested in patients with chronic LBP and the outcome concerned self-reported disability rather than pain [11, 16, 17]. When exclusively applied in patients with acute LBP or when using pain as outcome, the SBT predicted less accurately [16, 17]. Even prognostic tools that were specifically developed for acute LBP, such as the (short) Orebro Musculoskeletal Pain Questionnaire [12, 13], PICK-UP tool [14], as well as multiple other prediction models [18–22] demonstrated only acceptable predictive performance at best [16].

New research should therefore strive for better prognostic tools for acute LBP, which could be reached through including currently ignored predictors as well as repeated measurements over time (specifically in the first weeks to take into account the initial change [18, 23]). For optimal adoption in daily practice of such a new prognostic tool, it is conditional that it consists of only a limited number of predictors in order to minimize the burden for patients and clinicians, is integrated within an online clinical decision support system and is easy to interpret [24, 25]. The recently introduced artificial intelligence (AI)-based machine learning (ML) techniques have been suggested to be very promising and potentially able to result in a breakthrough in LBP (non-)recovery prediction [26, 27]. ML – in comparison to traditional regression analysis – is considered to be more flexible and pragmatic in handling complex datasets with large number of predictors (and their interactions), without strict rules regarding sample sizes and missing values [28].

The primary aim of this study is to develop and internally validate a prognostic ML model for predicting LBP non-recovery in patients with an acute episode of LBP. As secondary aims, we will compare the performance of this ML model with (i) current practice in physiotherapy (i.e., SBT and physiotherapists’ expectation), and (ii) a ‘traditional’ logistic regression model.

## Patients and methods

### Design

This is a prospective cohort study with a follow-up period of three months. No blinding of any measurement occurred during the study. This study is reported in accordance of the STROBE [29] (Additional file 1) and TRIPOD checklists [30] (Additional file 2).

The study was conducted in accordance with the Declaration of Helsinki and ethical guidelines of the HAN University of Applied Sciences. Ethical approval was received from the local ethical committee of the HAN University of Applied Sciences at 28–01-2019 (number: 141.01/19). All participants provided written informed consent. This study was funded by *Regieorgaan SIA* (PRJ006137). The funder played no role in the design of the study, collection, analysis, and interpretation of data and writing the manuscript.

### Setting

For patient inclusion, we recruited 99 Dutch physiotherapists in primary care to participate in our study for patient selection and inclusion, of which 64 did deliver one or more included patients. Physiotherapists could participate if they worked in a primary care setting and had experience in treating patient with LBP (i.e., ≥ one new LBP patient each week). Patient inclusion started from April 2019 and was intended to end at March 2020, but was prolonged until December 2020 due to the temporary closure of physiotherapy practices during the covid-19 lock-down.

### Participants

People with LBP were eligible if they met all of the following inclusion criteria:

- acute episode of LBP, which was operationalized as a recent onset (new) episode with duration of LBP symptoms ≤ one month;
- age between 18 and 85 years;
- informed consent.
  In addition, people were excluded if they met one of the following exclusion criteria:
- indication for a specific, patho-anatomical cause of LBP;
- not able to read and understand Dutch questionnaires.

### Sample size

In ML, a sample size calculation is generally not performed as there is no consensus regarding sample sizes for ML [28]. However, we aimed a priori at including at least 300 participants.

### Measurements

Participants received online (web-based or smartphone-based) questionnaires at baseline (T0) and at one (T1) and two weeks (T2), and three months follow-up (T3). If preferred by the participants, we provided questionnaires on paper.

#### Candidate predictors

Candidate predictors (see Table 1) have been selected based on the following criteria:

i. having a theoretical association with (non-)recovery of acute LBP, as reported in systematic reviews [2, 6–9], or consensus in an expert group of clinicians, researchers and patients on potential prognostic value of emerging factors;
  ii. being simple and reliable to measure in practice;
  iii. factors retrievable as a single item from validated questionnaires preferred over multi-item questionnaires (to minimize the burden).

**Table 1:** Overview of candidate predictors

	**Included based on:**				
	**Prognostic evidence**	**Expert opinion**	**Adopted from existing questionnaire**	**Time-points**	**Change T0-T2**
**Demographic factors**					
Age	X [6, 7]		n/a	T0	no
Gender	X [7]		n/a	T0	no
Educational level		X	n/a	T0	No
Other health issues	X [2, 7, 9]		STarT MSK item 7	T0	No
Shoulder and/or neck pain		X	STarT Back item 2	T0	No
Physical activity level		X	n/a	T0, T1, T2	yes
**Pain-related factors**					
Pain severity	X [7, 9]		NRS	T0, T1, T2	Yes
Frequency of previous LBP	X [7]		OMPQ item 11	T0	No
Disability of previous LBP episode		X	n/a	T0	No
Onset of LBP episode (sudden/ gradually)	X [7]		n/a	T0	No
Radiating pain in leg(s)	X [6, 7]		STarT Back item 1	T0	No
Disability	X [2, 7, 9]		STarT Back item 3, 4 and 9	T0, T1, T2	Yes
**Occupational factors**					
Work absenteeism	X [7]		n/a	T0, T3	No
Physically demanding work	X [6, 7]		OMPQ item 8	T0	No
Job satisfaction	X [6]		OMPQ item 17	T0	No
Work ability		X	WAI- Single item	T0	No
**Psychological factors**					
Psychological distress	X [6, 8, 9]		STarT Back item 6	T0, T1, T2	Yes
Depressive mood	X [7–9]		STarT Back item 8	T0, T1, T2	Yes
Fear of movement	X [2, 9]		STarT Back item 5	T0, T1, T2	Yes
Catastrophizing thoughts	X [8]		STarT Back item 7	T0, T1, T2	Yes
Pain coping	X [2, 7]		OMPQ item 12	T0, T1, T2	Yes
Recovery expectation	X [7, 9]		n/a	T0, T1, T2	Yes
Resilience		X	Vita-16	T0, T1, T2	Yes

*NRS* Numeric Rating Scale, *OMPQ* Orebro Musculoskeletal Pain Questionnaire, *STarT MSK* Keele STarT MSK Screening Tool, *STarT Back* Keele STarT Back Screening Tool, *WAI* Work Ability Index

After review of the literature and discussion with an expert group, most of the candidate predictors were considered stable over time and therefore only assessed at baseline, while only those that owere considered potentially modifiable or fluctuating, were also assessed at T1 and T2, to enable the calculation of change scores for the first 2 weeks. In case of missing values at T2, we used scores from T1, if available.

#### Outcomes

The primary outcome was LBP non-recovery at three months follow-up, defined as having at least mild pain (Numeric Rating Scale (NRS) score > 2 on a 10-point scale for pain severity in the past week), as previously proposed [31, 32] and applied by others (e.g. [5, 20],). The following operationalizations of LBP non-recovery were used as secondary outcome measures:

i. NRS > 1 for pain severity in past week;
ii. current pain not considered acceptable for the rest of their life (Pain Acceptability Symptom State (PASS));
iii. perceived recovery not reaching at least ‘better’ on Global Perceived Effect (GPE) scale.

#### Current practices

To explore the added value of our prognostic model for clinical practice, we also performed analyses with the two current practices for predicting LBP non-recovery in physiotherapy: SBT risk profiles (low vs. medium/high) and the physiotherapists’ expectation based on clinical expertise (recovered vs. not recovered in three months).

#### Treatment parameters

All patients were allowed to receive physiotherapy, as well as any other care. Physiotherapists registered the number of provided sessions, number of weeks of the treatment and the applied interventions (e.g., exercise therapy, mobilization), for each of their participants.

### Analysis

Baseline characteristics, outcomes and treatment parameters were descriptively analyzed (i.e., mean and standard deviation (SD) for continuous variables; numbers and percentages for categorical variables).

For our main objective, we used XGBoost [33] as this one appeared to be the most suitable Machine Learning (ML) method when considering our data and objective. Imputation of missing data is not necessary in this method. Technical specifications of the ML analysis are described in Additional file 3. In summary, we executed a fivefold cross-validation method [34], meaning that the dataset was split into 5 random sets of equal sample sizes, from which 4 sets (training sets) were used to train the algorithm and the fifth set (test set) was used to test this model. Each of the 5 random sets were used once as a test set, so this process was performed 5 times. In addition, the full process of splitting the dataset into 5 random sets was repeated 3 times, meaning that in total 15 cross-validated algorithms (i.e., 5 × 3) were developed, from which the average performance measures were reported. In this process, we used random oversampling in order to boost the underrepresented class and used grid search to optimize the parameters for each model. Recursive feature elimination of the cross-validated algorithms was applied, meaning that – based on the performance measures (which are mentioned below)—the least important predictor was removed from the model (roughly comparable to a backwards selection method from ‘traditional’ regression analysis), resulting in models with all potential predictors up to a 1-item model with only the most important predictor. From all of these models, we determined the ‘best’ performing one, i.e., combination of high predictive performance and low number of predictors (in order to facilitate its usage in clinical practice despite the time constraints of physiotherapists). Finally, this full cross-validation process was performed twice: (i) with baseline values only, and (ii) with baseline values plus week 0-week 2 change scores (in order to determine any added value of change scores for the predictive performance). Predictive performance was expressed by the Area Under the Curve (AUC; for discriminative performance) and the accuracy (i.e., fraction of true positive and true negative cases among the total number of cases). Two graphs were also made: Receiver Operator Curve (ROC) plot (for discriminative performance) and calibration plot (for calibration performance (‘goodness of fit’)).

For the secondary objectives, we first compared the performance of the final ML model with two current practices for predicting LBP (non-)recovery in physiotherapy, namely (i) the SBT risk profile classification (low vs. medium/high risk) and (ii) the physiotherapists’ expectation (recovery vs. non-recovery). For this purpose, two logistic regression models were developed: one with SBT risk profile and one with physiotherapists’ expectation as independent variables (both with recovery vs non-recovery as dependent variable). Second, we also compared the performance of the ML model with a ‘traditional’ (non-ML) logistic regression model using the same variables as used for the ML-model. A backward selection method (i.e., starting with all predictors in one model and then removing predictors one by one based on the largest *p*-value (if *p* ≥ 0.05)) was applied resulting in a final model (with predictors with *p* < 0.05 only). This final model was subsequently internally validated by bootstrapping (i.e., 250 samples, with shrinkage factor of 0.9924). Prior to the logistic regression analyses, collinearity between predictors was checked, and in case of a correlation coefficient > 0.9 between two predictors, one of both were selected for our analysis based on clinical application. The linearity assumption for the association between continuous predictors and the outcome was explored by checking linearity in this association across the four quartiles of the predictors. The logistic regression analysis was based on complete cases (i.e., cases with missings removed). As a substantial proportion of the sample (22%) did not have a job and we did not want these participants to be excluded from the analysis, we removed the work-related variables absenteeism due to LBP, physically demanding work, job satisfaction and work ability from this analysis (as these were only measured in people with a job). The predictive performance of these three logistic regression models were expressed by the AUC with ROC plot and accuracy, which could both be compared with the final ML-model, in addition to a Hosmer & Lemeshow test for the ‘goodness of fit’ (calibration) of the logistic regression models.

ML analyses were performed in Python version 3.7.4, libraries scikit-learn v0.23.2 and XGBoost v1.1.1; logistic regression analyses in SPSS version 25 and R version 4.0.3.

## Results

**Fig. 1:**
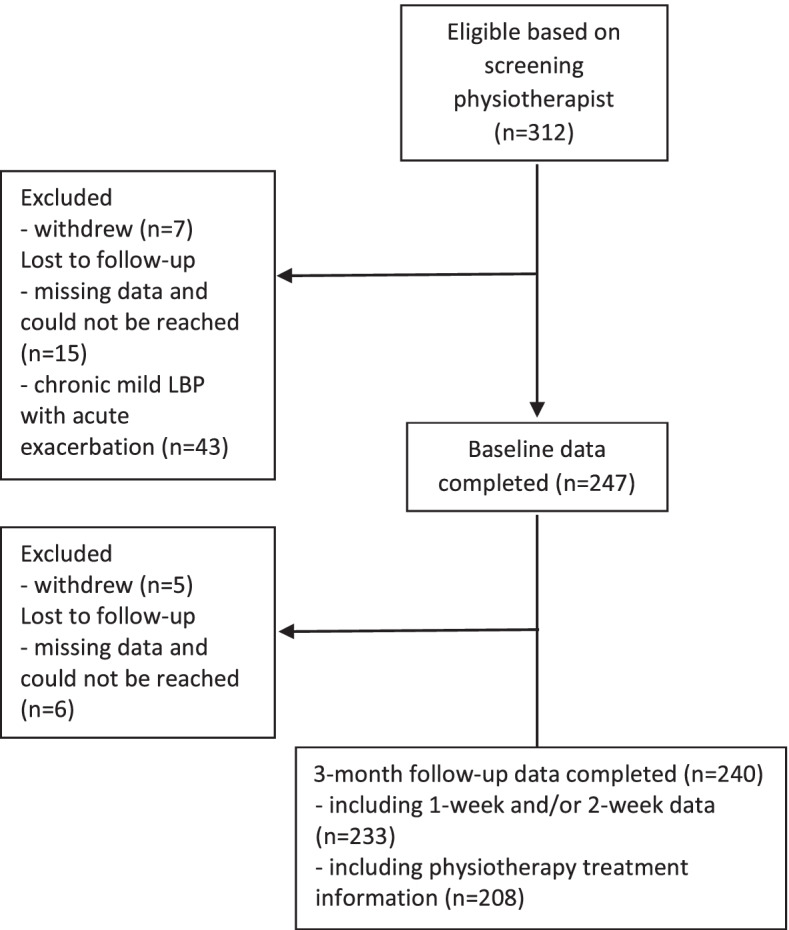
Flow chart of study inclusion

A total of 312 patients with acute LBP was included, from which we obtained baseline data of 247 (79%), both baseline and follow-up data from 240 (77%) and treatment parameters (i.e., duration, content) from 208 (67%). Figure 1 shows the flow chart of this inclusion, including reasons for non-participation and drop-out.

Baseline characteristics of our total sample and of the subsamples ‘LBP recovery’ and ‘LBP non-recovery’ (based on our primary outcome) are described in Table 2. Our total sample (*n* = 247) consisted of 41% females, the mean age (± SD) was 49 ± 15 years and mean LBP severity on a 0–10 scale at baseline was 6.9 ± 1.7. Based on the SBT, subjects could be labeled as ‘low risk profile’ in 46%, ‘medium risk profile’ in 46% and ‘high risk profile’ in 9%. The physiotherapists predicted recovery within 3 months in 96% of their patients, while only in 4% they predicted non-recovery. Work absence due to LBP was reported in 23% of the total sample at baseline, which reduced to 4% at 3 months follow-up. The study participants received on average 3.7 ± 2.2 physiotherapy sessions during their follow-up period. Most applied interventions in this treatment were education/advice (98%) active mobilization (68%) and manual therapy (64%).

**Table 2:** Baseline characteristics for total sample and subsamples ‘LBP recovery’ and ‘LBP non-recovery’

	**Missings**	**Total sample (*n* = 247)**	**‘LBP recovery’ (*n* = 126**^**a**^**)**	**‘LBP non-recovery (*n* = 114**^**a**^**)**
**Demographic factors**				
Age	*n* = 1	49 ± 15	49 ± 15	50 ± 15
Gender (female)	*n* = 0	102 (41%)	51 (41%)	48 (42%)
Educational level (low^b^)	*n* = 0	147 (60%)	70 (56%)	72 (63%)
Other health issues (yes)	*n* = 0	56 (23%)	27 (21%)	29 (25%)
Physical activity level (0–10; 0 = not active; 10 = very active)	*n* = 1	6.1 ± 2.0	6.1 ± 2.0	6.0 ± 2.0
**Pain-related factors**				
Pain severity in past week (0–10; 0 = no pain at all; 10 = worst pain imaginable)	*n* = 0	6.9 ± 1.7	6.8 ± 1.9	7.0 ± 1.4
Frequency of previous LBP in past 3 months (0–10; 0 = never; 10 = always)	*n* = 0	3.7 ± 3.0	2.8 ± 2.7	4.6 ± 3.0
Disability of previous LBP episode (Likert scale)	*n* = 0			
Not applicable (no previous episode)		42 (17%)	30 (24%)	11 (10%)
Not disabling		4 (2%)	3 (2%)	1 (1%)
Somewhat disabling		33 (13%)	22 (18%)	10 (9%)
Moderately disabling		71 (29%)	32 (25%)	36 (32%)
Very disabling		83 (34%)	33 (26%)	48 (42%)
Extremely disabling		14 (6%)	6 (5%)	8 (7%)
Type of onset:	*n* = 0			
Sudden		161 (65%)	93 (74%)	66 (58%)
Gradual		86 (35%)	33 (26%)	48 (42%)
**Work-related factors**				
Paid job (yes)	*n* = 0	193 (78%)	98 (78%)	88 (77%)
Current absenteeism due to LBP		45 (23%)	22 (22%)	23 (26%)
Physically demanding work (0–10; 0 = not heavy/ monotonous; 10 = extremely heavy/ monotonous)	*n* = 54^c^	4.8 ± 2.7	4.4 ± 2.7	5.3 ± 2.5
Job satisfaction (0–10; 0 = not satisfied at all; 10 = very satisfied)	*n* = 54^c^	7.3 ± 1.7	7.4 ± 1.8	7.2 ± 1.6
Work ability (0–10; 0 = not able to work; 10 = lifetime best)	*n* = 54^c^	6.5 ± 2.0	6.6 ± 1.9	6.4 ± 2.0
**Psychological factors**				
Pain coping (0–10; not capable to reduce pain at all; 10 = highly capable to reduce pain)	*n* = 0	5.8 ± 1.9	6.0 ± 2.0	5.7 ± 1.9
Recovery expectation (0–10; 0 = not likely to recover in 3 months; 10 = very likely to recover in 3 months)	*n* = 3	7.7 ± 2.3	8.2 ± 2.1	7.0 ± 2.4
Resilience (ability to recover after difficulties)	*n* = 0			
Always		54 (22%)	36 (29%)	18 (16%)
Almost always		85 (34%)	52 (41%)	29 (25%)
Mostly		67 (27%)	29 (23%)	36 (32%)
Regular		20 (8%)	4 (3%)	16 (14%)
Sometimes		4 (2%)	1 (1%)	2 (2%)
Occasionally		13 (5%)	2 (2%)	11 (10%)
Rarely		4 (2%)	2 (2%)	2 (1%)
STarT Back items:	*n* = 0			
1. Radiating pain in leg(s) (yes)		95 (38%)	43 (34%)	48 (42%)
2. Shoulder/neck pain (yes)		105 (43%)	46 (37%)	57 (50%)
3. Walking slowly (yes)		123 (50%)	45 (51%)	57 (50%)
4. Dressing slowly (yes)		181 (73%)	100 (79%)	78 (68%)
5. Fear of movement (yes)		68 (28%)	35 (28%)	32 (28%)
6. Psychological distress (yes)		82 (33%)	40 (32%)	41 (36%)
7. Catastrophizing (yes)		14 (6%)	4 (3%)	8 (7%)
8. Depressive mood (yes)		96 (39%)	44 (35%)	50 (44%)
9. Bothersomeness (very much/extreme)		138 (56%)	77 (61%)	59 (52%)
STarT Back risk profile:	*n* = 0			
Low risk		113 (46%)	60 (48%)	48 (42%)
Medium risk		113 (46%)	57 (45%)	55 (48%)
High risk		21 (9%)	9 (7%)	11 (10%)
Physiotherapists’ expectation:	*n* = 1			
Recovery within 3 months		237 (96%)	123 (98%)	107 (95%)
Non-recovery within 3 months		9 (4%)	3 (2%)	6 (5%)
**Received physiotherapy-treatment**		***N* = 208**	***N* = 102**	***N* = 99**
Number of sessions	*n* = 39	3.7 ± 2.2	3.5 ± 1.9	4.0 ± 2.4
Number of weeks	*n* = 48	3.9 ± 3.1	3.5 ± 2.5	4.4 ± 3.6
Applied interventions:	*n* = 39			
Patient education/advice		203 (98%)	98 (96%)	98 (99%)
Exercise therapy		101 (51%)	46 (45%)	52 (53%)
Manual therapy		134 (64%)	71 (70%)	57 (58%)
Active mobilization		142 (68%)	67 (66%)	73 (74%)
Passive mobilization		77 (37%)	40 (39%)	37 (37%)
Massage		83 (40%)	43 (39%)	43 (43%)
Dry needling		16 (8%)	6 (6%)	8 (8%)
Other		25 (12%)	1 (1%)	0 (0%)
Referral to other discipline		2 (1%)	0 (0%)	2 (2%)

^a^only those included with data on outcome measure (i.e., 240 of the 247 participants)

^b^low level of education defined as primary/secondary school/ post-secondary vocational education as highest degree

^c^not applicable (no paid job)

Around half (47%) of the participants could be defined as ‘LBP non-recovery’, with the other half (53%) defined as ‘LBP recovery’, when using the cut-off of our primary outcome. With the cut-offs of our secondary outcome measures, non-recovery proportions varied widely across outcomes, ranging from 17% (for GPE) to 64% (for NRS > 1) (Table 3). As shown by Table 2, the ‘LBP recovery’ subsample (*n* = 126) differs from the ‘LBP non-recovery’ subsample (*n* = 114) on frequency of previous LBP episodes in past 3 months on 0–10 scale (2.8 ± 2.7 for ‘LBP recovery group’ vs. 4.6 ± 3.0 for ‘LBP non-recovery group’), disability of previous LBP episode (very to extremely disabling in 31% vs. 49%), type of onset of current LBP episode (sudden onset in 74% vs 58%), patient’s recovery expectation on 0–10 scale (8.2 ± 2.1 vs. 7.0 ± 2.4) and resilience ((almost) always being able to recover after difficulties in life in 70% vs. 41%).

**Table 3:** Outcome measures

	**Baseline (T0) (*n* = 247)**	**2-week FU (T2) (*n* = 233)**	**3-month FU (T3) (*n* = 240)**
**Primary outcome measure**			
Pain severity			
Mean ± SD	6.9 ± 1.7	3.7 ± 2.4	2.1 ± 2.3
LBP recovery (NRS ≤ 2)	4 (2%)	48 (21%)	126 (53%)
LBP non-recovery (NRS > 2)	243 (98%)	185 (79%)	114 (47%)
Missing	*n* = 0	*n* = 14	*n* = 7
**Secondary outcome measures**			
Pain severity			
LBP recovery (NRS ≤ 1)	2 (1%)	16 (7%)	87 (36%)
LBP non-recovery (NRS > 1)	245 (99%)	217 (93%)	153 (64%)
Missing	*n* = 0	*n* = 14	*n* = 7
Pain acceptance			
Yes	19 (8%)	96 (44%)	197 (82%)
No	228 (92%)	123 (56%)	43 (18%)
Missing	*n* = 0	*n* = 28	*n* = 7
Global perceived effect			
(Very) much improved	n/a	151 (69%)	199 (83%)
Not much improved		68 (31%)	41 (17%)
Missing		*n = 28*	*n = 7*

From all ML-models, the 3-item model was the best performing model (i.e., best predictive value with least number of factors). This final model, consisting of resilience (6-point Likert scale), disability of previous LBP episode (6-point Likert scale) and patient’s recovery expectation (0–10 scale), demonstrated an AUC of 0.66 and an accuracy of 63%. Models that also included change scores of predictors for the first two weeks showed no substantial better performance compared to those without change scores. Table 4 shows the included predictors and the model’s performance parameters of models with one to ten predictors, based on the RFE method. Due to the tree-based algorithm method, regression estimates of the factors and a regression equation cannot be presented.

**Table 4:** Performance parameters of ML models with primary outcome measure for LBP non-recovery (NRS > 2) (with final 3-item model in bold)

	**AUC (95% CI)**	**Accuracy**
1. 1-item model: resilience	0.61 (0.53–0.69)	58%
2. 2-item model: 1 + patient’s recovery expectation	0.65 (0.55–0.70)	62%
**3. 3-item model: 2 + disability previous LBP episode**	**0.66 (0.56–0.70)**	**63%**
4. 4-item model: 3 + bothersomeness (SBT item 9)	0.65 (0.55–0.70)	61%
5. 5-item model: 4 + physically demanding work	0.64 (0.55–0.69)	62%
6. 6-item model: 5 + work absenteeism	0.64 (0.56–0.70)	60%
7. 7-item model: 6 + frequency previous LBP episodes	0.64 (0.56–0.70)	59%
8. 8-item model: 7 + physical activity	0.63 (0.55–0.70)	60%
9. 9-item model: 8 + work ability	0.64 (0.54–0.69)	61%
10. 10-item model: 9 + pain severity	0.63 (0.55–0.69)	59%

*AUC* Area under the curve, *CI* Confidence interval

The two current practices for predicting LBP recovery in physiotherapy were found to predict poorly, with AUC of 0.53 and accuracy of 53% for SBT risk profiles (low vs medium/high risk profile) and AUC of 0.53 and accuracy of 54% for physiotherapists’ expectation (see Table 5). Similar results were found when using SBT risk profiles as an ordinal variable (low vs. medium, low vs. high risk) instead of a dichotomous variable (low vs. medium/high risk).

**Table 5:** Performance parameters of model with (a) SBT risk profile or (b) physiotherapists’ expectation as predictor with primary outcome measure for LBP non-recovery (NRS > 2)

	**OR (95% CI)**	**AUC (95% CI)**	**Accuracy**	**Hosmer & Lemeshow test *p*-value**
**Model (a) SBT risk profile**				
Low (reference) vs medium/high risk	1.25 (0.75–2.08)			
Model summary		0.53 (0.45–0.60)	53%	n/a^a^
**Model (b) Physiotherapists’ expectation**				
Expectation of recovery (reference) vs. expectation of no recovery	2.27 (0.56–9.09)			
Model summary		0.53 (0.46–0.61)	54%	n/a^a^

*OR* Odds ratio, *CI* Confidence interval, *AUC* Area under the curve, *n/a* not applicable

^a^test was not possible as model consisted of one dichotomous variable

The ‘traditional’ logistic prediction modelling with backwards selection resulted in a 2-item model consisting of resilience (6-point Likert scale) and frequency of previous LBP episodes (0–10 scale). Regression estimates of the included variables are described in Table 6. The model demonstrated comparable or even slightly better performance than the ML-model with an AUC of 0.71 (95% CI: 0.65–0.78) and an accuracy of 68%, and appeared to have a good fit (Hosmer & Lemeshow test with *p*-value > 0.05). The regression equation following this model is: Y = -1.823 + (0.3594176 * resilience) + (0.1752032 * frequency of previous LBP episodes).

**Table 6:** Performance parameters of final, internally validated logistic regression model with primary outcome measure for LBP non-recovery (NRS > 2)

	**OR (95% CI)**	**AUC (95% CI)**	**Accuracy**	**Hosmer & Lemeshow test *p*-value**
Resilience	1.44 (1.15–1.80)			
Frequency previous LBP episodes	1.19 (1.08–1.31)			
Model summary		0.71 (0.65–0.78)	68%	0.536

*OR* Odds ratio, *AUC* Area under the curve, *CI* Confidence interval

**Fig. 2:**
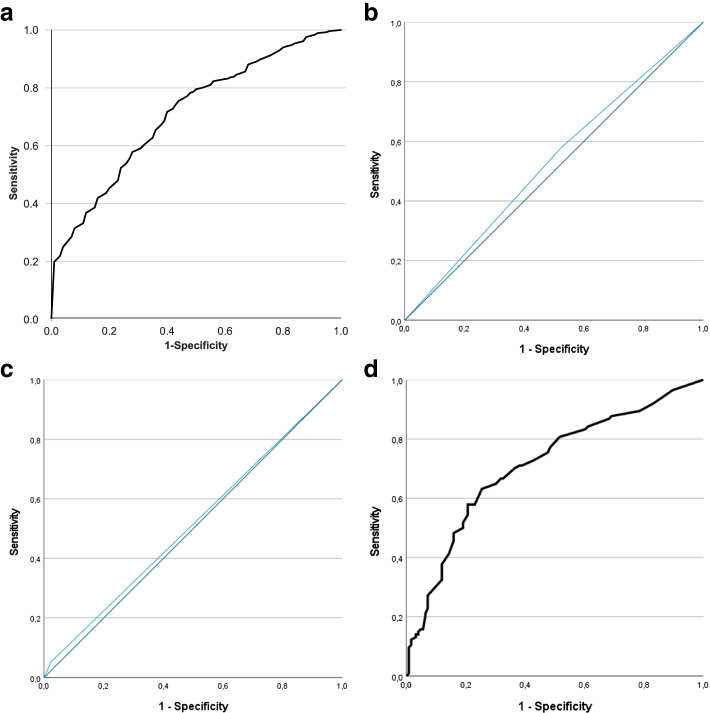
ROC-curves of (**a**) final ML-model, (**b**) SBT low vs medium/high risk profile, (**c**) physiotherapists’ expectation of recovery and (**d**) final logistic regression model

**Fig. 3:**
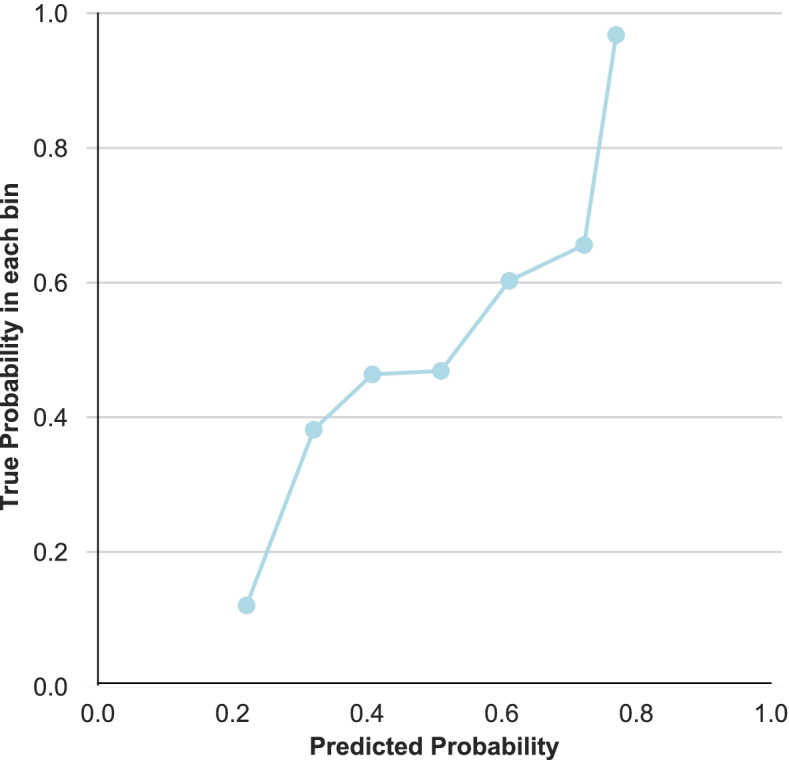
Calibration plot of final ML-model

Figures 2a-d display the ROC-curves for the 4 models (ML-model, SBT risk profile, physiotherapists’ expectation and logistic regression model), whereas Fig. 3 shows the calibration plot of the ML-model.

## Discussion

We developed a 3-item ML model consisting of 3 relatively new factors (resilience, disability of previous LBP episode and patient’s recovery expectation). This model predicted LBP non-recovery in two thirds of patients with acute LBP, which can be considered only acceptable and no better than a ‘traditional’ regression model. On the other hand, our models performed better than current practice in physiotherapy. Therefore, both models have the potential of integration in a clinical decision support system, to support personalized care in acute LBP. However, external validation should be performed first.

### Comparison with literature

Both the ML model and the logistic regression model showed predictive performances comparable to previously reported models in acute LBP (i.e., AUC around 0.6–0.7) [2, 14, 16–22]. In both models, initial change in prognostic factors (between week 0 and 2) had no added value, which is in contrast to previous research [18, 23, 35]. Possibly, the time window of two weeks was too short to have prognostic value in our study. A second unexpected finding was that the performance of the model from advanced ML was not superior to ‘traditional’ logistic regression analysis. Similar findings have also been reported in other studies comparing ML with logistic regression (e.g. [36]), which emphasize that overly high expectations for ML need to be nuanced.

The predictors for LBP persistence in our final ML model were resilience, disability of previous LBP episode and patient’s recovery expectation, while the logistic regression model consisted of resilience and frequency of previous LBP episodes. Although our models show some overlap with existing prognostic models for acute LBP [2–9, 14, 16–23], it is striking that these existing models mostly contain different predictors [2–9, 14, 16–23]. Even our ML-model and logistic regression model partly differ in their predictors. This illustrates that prognostic research highly depends on study context (e.g., country, health care setting, case-mix, inclusion and exclusion criteria), study characteristics (e.g., predictors included in the studies, definitions of (non-)recovery), as well as on applied analytical approach (e.g., ML, ‘traditional’ logistic regression). Prognostic models and tools should therefore be strictly applied in the context that they were developed in.. Moreover, the wide fluctuations in predictors across prognostic models also emphasize the importance of external validation and replication of these models, prior to implementation in clinical practice.

As far as we know, resilience (i.e., being able to (mentally) recover from difficulties in life) has not yet been frequently used in prognostic LBP research (e.g. [37, 38]), with no studies in acute LBP. We were surprised that while resilience was found to be a prognostic factor, none of the well-accepted and frequently reported psychological factors (e.g., psychological distress [6, 8, 9, 19], depressive mood [2, 7–9, 14, 18, 19], fear of movement [2, 9] or catastrophizing [8, 20, 22]) did. One explanation could be that ‘negative’ psychological factors may play a more dominant and evolving role in the subacute or chronic rather than the acute phase, in contrast to resilience that might be (even more) important in the acute phase. Another explanation could be that the psychological factors were assessed by single items in our study, therefore not fully covering the full construct (although this also applies to resilience). Our results may therefore indicate that resilience should be considered as a new and more positively oriented psychological factor in LBP persistence. We recommend that future studies will include resilience in their analyses in order to replicate our findings. In addition, new studies should explore whether resilience can be modified by treatment and therefore a potential factor in preventing LBP chronicity. This also counts for recovery expectation, which was a prognostic factor in our ML-model and is considered to be potentially modifiable.

Our ML model outperformed current practices in physiotherapy (i.e., SBT and clinician’s expectation). Also other studies found that neither the widely used SBT [16, 39–41] nor a health care provider [39] can accurately predict LBP non-recovery, although some other studies showed good predictive value for the SBT [19] and the health care provider [22]. As a first explanation for the poor predictive performance of the SBT, it should be noted that this tool was not developed for the purpose to predict LBP recovery but to distinguish risk profiles to provide stratified care, and not for patients the acute phase. A second possible explanation is that the SBT only consists of modifiable factors, thereby missing important prognostic factors that are non-modifiable (e.g., frequency of previous episodes). A third explanation might be that the patient’s clinical status (and thereby the SBT item scores as well) may fluctuate easily in the first days after episode onset, and that the predictive performance of the SBT increases when being assessed later in the (sub)acute phase [41].

### Relevance for clinical practice

Our finding of the SBT and physiotherapists’ expectation not being predictive suggests that health care providers should be cautious in relying on the, at least in the Netherlands, widely used SBT or their own expertise in their prognosis in patients with acute LBP. Ideally, as an alternative, a prognostic tool that is specifically developed for this purpose and has been externally validated should be used. Such a tool, when integrated in a clinical decision support system, can be expected to facilitate providing a realistic prognosis and a data-driven, personalized treatment. If the prognosis is favorable, a patient could be directly reassured and unnecessary care possible prevented. If the prognosis is unfavorable, a treatment targeting potentially modifiable predictors (e.g., patient’s recovery expectation, resilience) may need to be directly applied. Based on our finding that change scores in the first two weeks did not improve the prediction, this tool could be used immediately during the intake, without waiting for the initial change in symptoms.

### Future research

The internally validated ML-model and logistic regression model should first be externally validated, before implementation in clinical practice could be considered. Future research should also focus at determining the added value of our model(s) embedded in a clinical decision support system on clinical outcomes. Our finding of resilience as emerging prognostic factor needs replication, as we were the first to report this. Finally, future studies may clarify whether resilience and recovery expectations can be modified by interventions, in order to prevent LBP chronicity.

### Limitations and strengths

We need to acknowledge the following limitations of our study. First, our sample size of 247 is relatively low for a prognostic study and lower than intended. However, in ML a large sample size is not considered as crucial as in a ‘traditional’ epidemiologic study. Second, there is a risk of overfitting and it should be noted that none of the models has been externally validated in other samples. The prognostic models and tools from our study should therefore not yet be implemented in clinical practice. Third, we initially included also chronic LBP patients with mild symptoms that experienced a recent (≤ 1 month) exacerbation, similar as Jellema et al [22] did. However, we decided to exclude them (*n* = 47, Fig. 1) from the analysis in order to have a ‘pure ‘ acute LBP cohort that can be more easily interpreted. Due to their chronic pain it was no reasonable to expect this subgroup would reach the outcome of having two or less points on a 10-point NRS for pain severity in the past week. We also analyzed our data including this subgroup of chronic patients with mild symptoms but found no differences in results except for belonging to this subgroup being an predictor as expected (data not shown). Forth, as a secondary objective, we compared ML with ‘traditional’ logistic regression analysis, but this comparison was affected by some differences in methodology (e.g., all cases included in ML vs. only complete cases (i.e., removal of work-related variables and of cases with missings) in logistic regression analysis). Fifth, our study is restricted to patient-reported factors, while ignoring other potentially important factors (e.g., inflammation, pain sensitization, genetics). Sixth, all participants received a physiotherapy treatment (on average four sessions). Although this treatment could theoretically have influenced the course of symptoms, this impact can be expected to be minimal, as physiotherapy has been found to be ineffective in the acute phase of LBP [42–44]. Seventh, we would ideally have compared the predictive performance of our new models with a ‘gold standard’. However, such a gold standard for predicting LBP (non-)recovery does not yet exist. Therefore, we used current practice (SBT and physiotherapists’ expectation) as the best available comparison, which also enabled us to explore the potential added value of our prognostic models when used in clinical practice. Eight, our dataset is limited to a 3 month follow-up period, while data from a longer time frame (e.g., 6 or 12 months) would have enabled us to verify how many people that developed persistent LBP in 3 months recovered soon afterwards. On the other hand, as chronic LBP is mostly defined as LBP for 3 months or longer, the 3 month time-point can be considered appropriate for our study aim.

The major strengths of our study are that we included a complete set of all patient-reported, prognostic factors that have been previously identified [2, 6–9], supplemented by some emerging factors, and that we determined the added value of our model over current practice methods for estimating the prognosis in acute LBP.

## Conclusions

We developed two prognostic models containing partially different predictors, with acceptable performance for predicting (non-)recovery in patients with acute LBP, which was better than current practice (i.e., SBT and physiotherapists’ expectation). Both models have the potential of integration in a clinical decision support system, to facilitate data-driven, personalized treatment of acute LBP, but needs external validation first.

## Supplementary Information

**Additional file 1.** Strobe checklist.

**Additional file 2.** TRIPOD checklist.

**Additional file 3.** Specifications ofML-analysis.

## Data Availability

The data will be made available upon reasonable request.

## Abbreviations

AI: Artificial intelligence
AUC: Area under the curve
GPE: Global perceived effect
ML: Machine learning
NRS: Numeric rating scale
LBP: Low back pain
PASS: Pain Acceptability Symptom State
SBT: STarT Back screening Tool
