# Supplementary material for: Development and internal validation of a machine learning prediction model for low back pain non-recovery in patients with an acute episode consulting a physiotherapist in primary care
Source: BMC Musculoskelet Disord. 2022 Sep 3;23:834. doi: 10.1186/s12891-022-05718-7 (PMC9440317; doi:10.1186/s12891-022-05718-7)
Supplement: Supplementary file 3 — Additional file 3. Specifications ofML-analysis. [file 12891_2022_5718_MOESM3_ESM.docx]

**Supplementary file 3: Specifications of ML-analysis**

# Phase 1 – Exploration

In the initial phase we used a subset of the data to explore the data and find promising models:

- Data: first 110 patients that completed all surveys
- Models with default parameters from sklearn package v0.23.2:
  - XGBoost
  - K Nearest Neighbors (K=3)
  - Linear Support Vector Classifier
  - Logistic Regression
  - Decision Tree Classifier
  - Random Forest Classifier
  - MLP Classifier (Neural Network)
  - Adaboost Classifier
  - Multionomial Naive Bayes Classifier
- Oversampling in order to deal with imbalance in data:
  - None
  - Random
  - SMOTE

From this comparison we concluded that XGBoost classifier (and also Random oversampling) was most promising, but there were no large differences between models.

# Phase 2 - Tuning Details

The XGBoost classifier was further tuned. The implementation from sklearn, XGBClassifier was used with a gbtree base learner. Tuning was done on a model using all features. The following parameters were tuned using a cross validated grid search with 10 folds and optimized for accuracy:

- Minimum loss required to make a split: gamma = [0.1, 0.2, 0.3, 0.4 0.5, 0.6]
- Number of trees: n_estimators = [100, 200, 300, 400, 500]
- Learning_rate = [0.01, 0.1, 0.2, 0.3, 0.4]
- Maximum depth of a tree: max_depth = [2,3,4,5,6,7,8]

The optimal combination in this grid search was:

- Gamma: 0.5
- Learning_rate: 0.3
- Max_depth: 3

The number of trees did not have influence on the results and was kept at the default of 100. These parameters were used in the remainder of the experiments.
